# Co-occurrence of resistance genes to antibiotics, biocides and metals reveals novel insights into their co-selection potential

**DOI:** 10.1186/s12864-015-2153-5

**Published:** 2015-11-17

**Authors:** Chandan Pal, Johan Bengtsson-Palme, Erik Kristiansson, D. G. Joakim Larsson

**Affiliations:** Department of Infectious Diseases, Institute of Biomedicine, Sahlgrenska Academy, University of Gothenburg, SE-413 46 Gothenburg, Sweden; Department of Mathematical Sciences, Chalmers University of Technology, SE-412 96 Gothenburg, Sweden

**Keywords:** Antibiotic resistance, Biocide resistance, Metal resistance, Bacterial genomes, Plasmids, Co-selection, Toxin-antitoxin

## Abstract

**Background:**

Antibacterial biocides and metals can co-select for antibiotic resistance when bacteria harbour resistance or tolerance genes towards both types of compounds. Despite numerous case studies, systematic and quantitative data on co-occurrence of such genes on plasmids and chromosomes is lacking, as is knowledge on environments and bacterial taxa that tend to carry resistance genes to such compounds. This effectively prevents identification of risk scenarios. Therefore, we aimed to identify general patterns for which biocide/metal resistance genes (BMRGs) and antibiotic resistance genes (ARGs) that tend to occur together. We also aimed to quantify co-occurrence of resistance genes in different environments and taxa, and investigate to what extent plasmids carrying both types of genes are conjugative and/or are carrying toxin-antitoxin systems.

**Results:**

Co-occurrence patterns of resistance genes were derived from publicly available, fully sequenced bacterial genomes (*n* = 2522) and plasmids (*n* = 4582). The only BMRGs commonly co-occurring with ARGs on plasmids were mercury resistance genes and the *qacE∆1* gene that provides low-level resistance to quaternary ammonium compounds. Novel connections between cadmium/zinc and macrolide/aminoglycoside resistance genes were also uncovered. Several clinically important bacterial taxa were particularly prone to carry both BMRGs and ARGs. Bacteria carrying BMRGs more often carried ARGs compared to bacteria without (*p* < 0.0001). BMRGs were found in 86 % of bacterial genomes, and co-occurred with ARGs in 17 % of the cases. In contrast, co-occurrences of BMRGs and ARGs were rare on plasmids from all external environments (<0.7 %) but more common on those of human and domestic animal origin (5 % and 7 %, respectively). Finally, plasmids with both BMRGs and ARGs were more likely to be conjugative (*p* < 0.0001) and carry toxin-antitoxin systems (*p* < 0.0001) than plasmids without resistance genes.

**Conclusions:**

This is the first large-scale identification of compounds, taxa and environments of particular concern for co-selection of resistance against antibiotics, biocides and metals. Genetic co-occurrences suggest that plasmids provide limited opportunities for biocides and metals to promote horizontal transfer of antibiotic resistance through co-selection, whereas ample possibilities exist for indirect selection via chromosomal BMRGs. Taken together, the derived patterns improve our understanding of co-selection potential between biocides, metals and antibiotics, and thereby provide guidance for risk-reducing actions.

**Electronic supplementary material:**

The online version of this article (doi:10.1186/s12864-015-2153-5) contains supplementary material, which is available to authorized users.

## Background

Our ability to treat many bacterial infections is challenged globally by the emergence and spread of antibiotic resistant bacteria, largely driven by the selection pressure from antibiotics [1, 2]. In addition, antibacterial biocides and metals have the ability to co-select for antibiotic-resistant bacteria [3]. This can occur if a common resistance mechanism is shared by the biocide/metal and the antibiotic, or if the bacterium carries resistance or tolerance genes to both types of compounds [4, 5]. Currently, the understanding of which biocides and metals that are likely to co-select for resistance towards certain classes of antibiotics is limited, as is reliable systematic data on environments and bacterial taxa where risks for co-selection are high. However, the increasing number of sequenced bacterial genomes and plasmids has opened up the possibility to investigate the co-occurrence of biocide/metal resistance genes (BMRGs) and antibiotic resistance genes (ARGs) on a larger scale.

If resistance genes for both antibiotics and biocides/metals are physically located on the same plasmid, metal/biocide exposure can also promote horizontal gene transfer (HGT) of antibiotic resistance. Today, conjugative plasmids carrying ARGs have become a major health challenge in clinical settings [6]. In addition, environmental microbial communities maintain diverse collections of resistance genes which might be mobilized into pathogenic bacteria via HGT [7–11]. Therefore, understanding the associations between plasmid mobility and their potential for co-selection on a larger scale would be valuable to improve our ability to identify risk scenarios.

A variety of biocides and metals, at concentrations found in different natural and polluted environments, have the ability to co-select for antibiotic-resistant bacteria and resistance plasmids [12]. Thus, it is possible that opportunities for co-selection are widespread across environments, taxonomic groups and different types of biocides and metals [3]. Still, there are most likely environments, taxa and co-selective agents associated with considerably higher risks. Compilation of data that enables the detection of common patterns on co-selection ability is, however, lacking. Hence, there is a need for systematic investigation of co-selective potential for different metals and biocides across environments and bacterial taxonomic groups, which could be the potential sources or routes for transmission of ARGs across ecological boundaries and eventually to pathogens.

Toxin-antitoxin (TA) systems stabilize plasmids in their hosts by killing daughter cells that do not inherit the plasmids [13]. Thus, plasmids with both ARGs and BMRGs (i.e. with co-selection potential) together with toxin-antitoxin systems would likely be more persistent, also in the absence of selection pressure, than would resistance plasmids lacking a toxin-antitoxin system. It is not known how often resistance genes and toxin-antitoxin systems occur together on plasmids, a knowledge that would add to our understanding of risks.

The overall aim of this study was to enhance our understanding of the co-selective potential between metals/biocides and antibiotics based on known co-localization of resistance genes in bacteria. We have therefore used a large-scale bioinformatic approach based on analysing publicly available, completely sequenced bacterial genomes and plasmids available in the NCBI repository, and investigated the co-occurrence of resistance genes, allowing us to identify combinations of genes, and hence indirectly, selective agents, where risks for co-selection are apparent. Furthermore, we have used information on the isolation source of all bacterial strains to discern if there are environments and certain bacterial taxa in which the co-selection potential is particularly high. Finally, we have investigated to what extent plasmids that carry both BMRGs and ARGs are conjugative and/or carry toxin-antitoxin systems.

## Methods

### Data collection and pre-processing

A dataset of 2522 publicly available completely sequenced bacterial genomes from 565 different genera (Additional file 1: Figure S1), comprising 2666 chromosomes and 1926 plasmids, were retrieved from NCBI bacterial genomes database [14] (ftp://ftp.ncbi.nlm.nih.gov/genomes/Bacteria/) on June 6 2014. The fully sequenced genomes contained less than half (42 %) of all completely sequenced plasmids. Therefore, in a separate dataset, the sequences of 1926 plasmids were expanded to 4582 plasmids hosted by 313 different bacterial genera (Additional file 1: Figure S2), by including other publicly available completely sequenced plasmids that were sequenced independently (58 %), from the NCBI plasmid genomes database [14] (ftp://ftp.ncbi.nlm.nih.gov/genomes/Plasmids/). GenBank annotation files of these chromosomes and plasmids were used in further analyses. Although these datasets constitute a large and diverse sample of bacterial species, they are not necessarily representative of the full microbial diversity.

Antibacterial biocide and metal resistance protein sequences were retrieved from the BacMet predicted database (version 1.1; http://bacmet.biomedicine.gu.se) [15]. As the main rationale of this study was to identify potential risk for dissemination of antibiotic resistance, we studied the co-occurrences of BMRGs with only horizontally transferrable ARGs. Therefore, we used the recently developed, manually curated Resqu database (version 1.1; http://www.1928diagnostics.com/resdb/) [16] containing only ARGs reported to have been horizontally transferred between at least two different bacterial species. Additionally, mobile genetic elements (MGEs) such as integron-associated integrase genes (*intI*) and *ISCR* transposases were also retrieved from the Resqu database. Metadata, such as isolation source of each bacterial isolate was collected from original literature, PATRIC [17] and the KEGG [18] databases (Additional file 2: Table S1). The 2522 bacterial genomes were categorised into twelve different environments based on their isolation source (Additional file 1: Figure S3; Additional file 3).

### Sequence analysis

The 2522 bacterial genomes and 4582 plasmids were subjected to similarity searches against the BacMet and Resqu databases using the ublast algorithm implemented in USEARCH (version 6.0.307) [19]. Both query and target coverage thresholds of 90 % over the entire sequence length and a sequence identity threshold of 90 % were set to retrieve only relevant matches (options “-ublast -accel 0.1 -id 0.9 -evalue 1.0e-10 -query_cov 0.9 -target_cov 0.9 -threads 16”). To detect potential conjugation systems on the plasmids, hidden Markov model (HMM) profiles of all well-described type 4 secretion systems [20, 21] were searched against the database of 4582 plasmid sequences using the hmmsearch program within HMMER (version 3.0) [22] (options "--cpu 16 --cut_tc --noali"). Similarly, genes representing toxin-antitoxin systems were detected on 4582 plasmids using HMM profiles of all well-described toxin-antitoxin systems collected from Pfam database (Additional file 1: Table S2) [23]. Finally, distribution patterns of bacteria and plasmids with co-selection potential were analysed across bacterial taxa and isolation environments.

### Co-occurrence analysis

A network connecting resistance genes found together on the same plasmid was built. Another network was built to connect resistance genes occurring together in the same bacterial strain irrespective of their location on chromosome or plasmid. Both networks were filtered for illustration purposes, so that BMRGs encountered together with ARGs on at least ten plasmids or in ten genomes were connected by an edge. In both networks, resistance genes that co-occurred together with integron-associated integrases or *ISCR* transposases at least ten times were also included. The networks were explored and visualized using Cytoscape (version 3.2.0) [24]. Heat maps were generated in R (http://www.r-project.org/) [25] using the package gplots [26] to show the co-occurrence patterns between BMRGs and ARGs that most frequently occur together in bacterial strains and only on plasmids.

### Statistical analysis

The frequency of ARGs was tested for significance between plasmids carrying BMRGs and without, using logistic regression adjusting for the impact of plasmid size. In genomes (i.e. strains) the association was tested using Fisher’s exact test. Furthermore, logistic regression was used to test the likelihood of plasmids carrying both ARGs and BMRGs being conjugative with adjustment for the effect of plasmid size. Wilcoxon rank sum tests were performed to test the difference in sizes between plasmids with and without co-selection potential, as well as between conjugative and non-conjugative plasmids. Finally, the frequency of toxin-antitoxin systems was tested for significance between resistance plasmids with different combinations of resistance genes and plasmids without resistance genes. R code for statistical analysis is available in Additional file 4.

The purpose of this study was not to identify overrepresentation of co-occurrences between pairs of known resistance genes (i.e. more than expected than by random chance), but simply to identify combinations that are common in sequenced bacteria. Therefore, common resistance genes are more likely to occur in such combinations, and thus we have chosen not to normalize for the abundance of individual resistance genes.

## Results

### ARGs are associated with BMRG-carrying bacteria

Eighty percent of the sequenced bacteria were derived from three phyla—Proteobacteria (46 %), Firmicutes (23 %) and Actinobacteria (11 %) (Additional file 1: Figure S1). One-third of the bacterial genomes contained at least one plasmid and 11 % of all bacterial genomes harboured resistance plasmids (Additional file 1: Table S3).

**Fig. 1:**
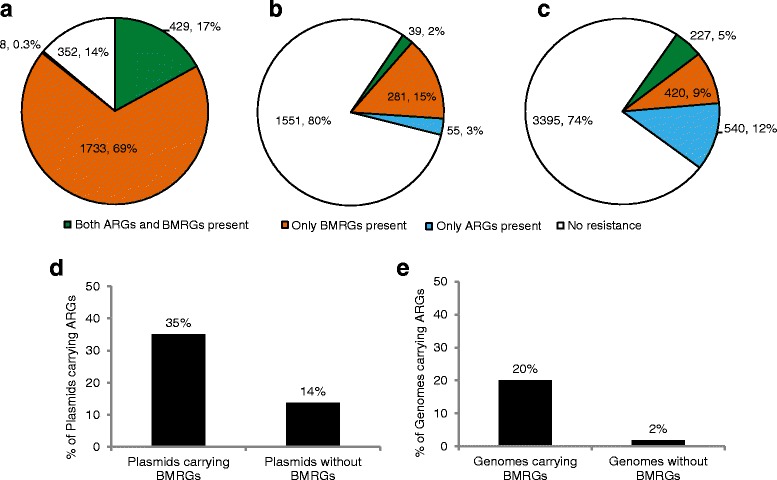
Overview of resistance information in genomes and plasmids, and relative proportions of ARG-carrying plasmids and genomes. Resistance gene profiles of **a** 2522 genomes irrespective of location on chromosomes or plasmids, **b** 1926 plasmids harboured by the 2522 genomes and **c** 4582 plasmids, respectively. **d** Relative proportions of ARG-carrying plasmids with or without BMRGs on the same plasmid, and **e** Relative proportions of ARG-carrying genomes with or without BMRGs in the same genomes, either on a plasmid or chromosome

Of the 4582 plasmids analysed, one-fourth carried resistance genes (Fig. 1c) and in nearly half of the cases just a single resistance gene (Additional file 1: Figure S4). Five percent carried at least one BMRG and one ARG (Fig. 1c). Notably, the frequency of ARGs was significantly higher on BMRG-carrying plasmids (35 %) than plasmids without any BMRGs (14 %) (Fig. 1d), even when adjusting for the plasmid size (logistic regression, *p* < 0.0001).

For genomes, 17 % of the bacterial strains carried both ARGs and BMRGs either on chromosomes or plasmids (Fig. 1a). The majority of the genomes (86 %) carried genes involved in biocide/metal resistance mechanisms. Similar to the situation with plasmids, genomes with BMRGs more frequently carried ARGs compared to those without BMRGs (20 % versus 2 %; Fisher’s exact test, *p* < 0.0001) (Fig. 1e).

### Heavy metal, sulfonamide, aminoglycoside and beta-lactam resistance genes are common

On plasmids, resistance genes towards sulfonamides, beta-lactams, aminoglycosides and tetracyclines were particularly common (Additional file 1: Figure S5a). Genes involved in tolerance/resistance mechanisms towards metals such as mercury, cadmium, arsenic and copper were frequently observed on plasmids, as well as the biocide resistance gene *qacE∆1,* which can confer low-level resistance towards multiple chemical classes of biocides [27] including quaternary ammonium compounds (QACs), biguanides, acridines, diamidines, xanthenes, and phenanthridines (Additional file 1: Figure S5b). MGEs were also common and *intI* and/or *ISCR* transposases were found in over 10 % and 7 % of the plasmids carrying ARGs and/or BMRGs, respectively.

On chromosomes, resistance genes towards macrolides, aminoglycosides, sulfonamides, tetracyclines, amphenicols and beta-lactams were the most frequently encountered ARGs (Additional file 1: Figure S5c), whereas for biocides and metals, genes involved in tolerance/resistance mechanisms towards copper, arsenic, chromium and biocide classes, such as peroxides, QACs and acriflavines were relatively prevalent (Additional file 1: Figure S5d). Notably, many of the genes encoding efflux pumps may have a much broader specificity than indicated by their annotation.

### Clinically important genera host the largest numbers of ARGs and BMRGs

**Fig. 2:**
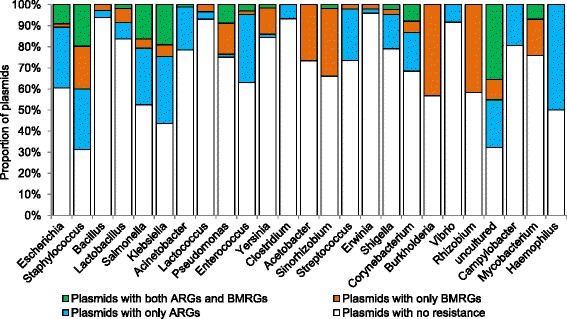
Overview of resistance information of 4582 plasmids found across bacterial genera. Only the 25 genera hosting the highest number of resistance plasmids are shown. Each individual section of a bar represents the proportion that carries different types of resistance genes. The green section in each bar indicates the number of plasmids that have both BMRGs and ARGs present on the same plasmid, and thus potential for co-selection

The 4582 plasmids belonged to bacteria from 313 genera, mainly from three phyla: Proteobacteria (47 %), Firmicutes (30 %) and Spirochaetes (9 %) (Additional file 1: Figure S2 and S6). ARGs were most common on plasmids hosted by *Providencia, Citrobacter, Klebsiella*, and *Enterobacter* (>3 ARGs/plasmids), while *Cupriavidus, Ralstonia* and *Cronobacter* carried plasmids with many BMRGs (>4 BMRGs/plasmid) (Additional file 1: Figure S7). A higher proportion of plasmids hosted by *Escherichia*, *Staphylococcus*, *Salmonella* and *Klebsiella* tended to carry both BMRGs and ARGs on the same plasmids than others (Fig. 2). Additionally, many plasmids with high potential for co-selection were found in uncultured bacteria. Plasmids usually carried more ARGs than BMRGs in many clinically important genera (Additional file 1: Figure S8). A notable exception was *Klebsiella,* on average harbouring more BMRGs than ARGs on their plasmids.

BMRGs were highly abundant on chromosomes, and some isolates of *Escherichia*, *Shigella*, *Klebsiella* and *Enterobacter* carried over 100 such genes in their genomes. Notably, some mobile ARGs were also relatively common (2–3 genes/genome) on the chromosomes of specific bacterial genera encompassing human pathogens such as *Acinetobacter*, *Staphylococcus* and *Enterococcus*.

### Distribution of ARGs and BMRGs across environments

**Fig. 3:**
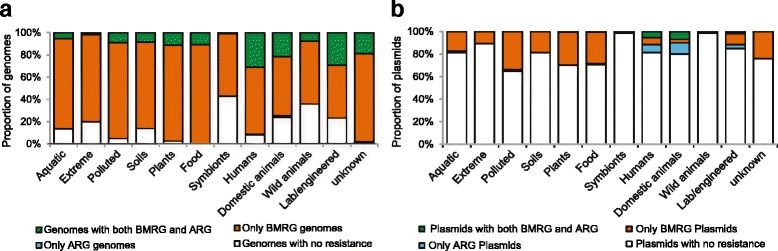
Overview of resistance information from genomes and plasmids from different environments. Resistance information from (**a**) 2522 completely sequenced bacterial genomes and (**b**) 1926 plasmids harboured by those genomes from different environments. Each individual section of a bar represents the proportion that carries different types of resistance genes. The green section of a bar represents the proportions of genomes (**a**) or plasmids (**b**) that have both ARGs and BMRGs present in the same strain (**a**) or on the same plasmid (**b**), thus representing the potential for co-selection

**Fig. 4:**
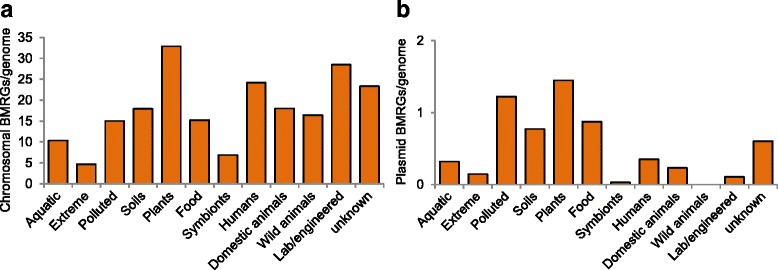
Relative abundance of biocide/metal resistance genes across environments. **a** Chromosomal and **b** Plasmid-borne biocide/metal resistance genes per genome

By studying genomes and plasmids isolated from multiple environments, we tried to identify risk environments for co-selection. In total, 17 % of all bacterial genomes (chromosomes and plasmids) carried both ARGs and BMRGs (Fig. 1a), whereas the corresponding figure was 31 % in those isolated from humans (Additional file 1: Table S4), followed by isolates from domestic animals (22 %). However, both types of genes were found together in genomes from several other isolation sources such as plants, food, polluted, aquatic and soil environments, although in lower proportions (Fig. 3a). When only plasmids from completed genomes (*n* = 1926) were considered, the majority were from humans or domestic animals (Fig. 3b). Importantly, a significant difference in prevalence of ARGs was observed between isolates from domestic and wild animals. Additionally, plasmids isolated from insect symbionts and wild animal sources carried almost no resistance genes at all. Furthermore, chromosomal BMRGs were ubiquitous across isolates from all environments (Fig. 4). Although for some isolates the environmental classification based on isolation source was partially a subjective decision and can be argued to be part of other similar environments, the major findings would not change substantially as the co-selection potential was mostly observed in a few specific and very distinct environments.

### The majority of plasmids carrying both ARGs and BMRGs are conjugative

Among 4582 plasmids, 20 % were found to be conjugative, 23 % mobilizable but lacking their own conjugation system and 57 % non-transmissible. Similar results were observed for the 1926 plasmids from only completely sequenced genomes (Additional file 1: Table S5).

**Fig. 5:**
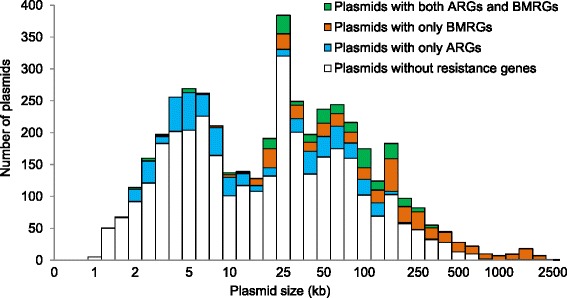
Resistance gene characteristics of 4582 plasmids in relation to their size. The individual sections of each bar represent the number of plasmids that carry different resistance gene types. The green section of each bar represents the number of plasmids with both ARGs and BMRGs on the same plasmid, and thus the potential for co-selection

**Fig. 6:**
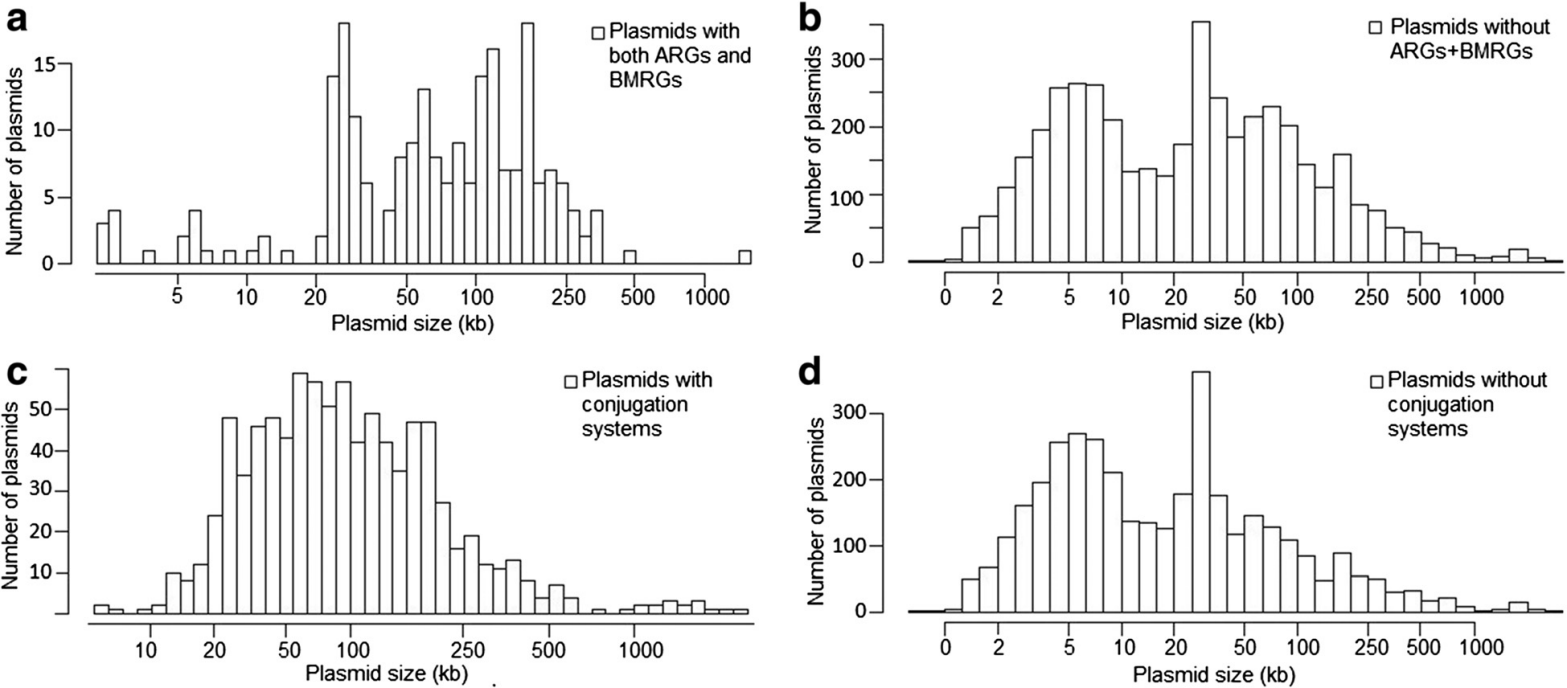
Distribution of plasmids with or without co-selection and mobility potential relative to their size. **a** Distribution of plasmids carrying both ARGs and BMRGs. **b** Distribution of plasmids carrying either ARGs or BMRGs or no resistance genes, thus lacking potential for co-selection of resistance based on co-occurrences. **c** Distribution of conjugative plasmids. **d** Distribution of non-conjugative plasmids (i.e. non-transmissible or mobilizable)

**Fig. 7:**
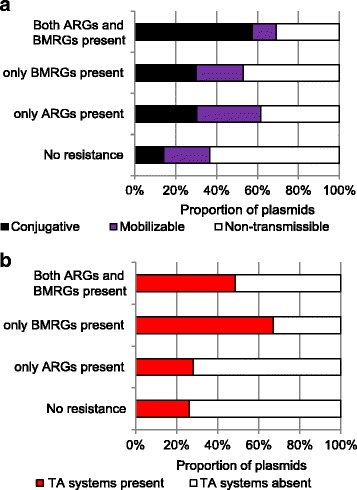
Overview of mobility potential and toxin-antitoxin systems on plasmids with different combinations of resistance genes. **a** Mobility potential (i.e. presence or absence of conjugations systems) in plasmids with different combinations of resistance genes. **b** Toxin-antitoxin (TA) systems in plasmids with different combinations of resistance genes

The 4582 plasmids ranged in size from less than 1 kb to over 2500 kb. Therefore we investigated whether there is an association between plasmid size, the presence of both BMRGs and ARGs on the same plasmid and their mobility potential. The great majority of the small resistance plasmids (<10 kb) carried only ARGs, but not BMRGs. Some resistance plasmids with a size over 20 kb carried both ARGs and BMRGs on the same plasmid, whereas plasmids over 250 kb generally did not (Fig. 5). Unsurprisingly, plasmids with both ARGs and BMRGs tended to be larger than those without (median sizes of 76 kb and 26 kb, respectively; Wilcoxon rank sum test, *p* < 0.0001) (Fig. 6a and b). Conjugative plasmids were larger than non-conjugative (both mobilizable and non-transmissible) plasmids (median sizes of 90 kb and 15 kb, respectively; Wilcoxon rank sum test, *p* < 0.0001) (Fig. 6c and d). A large proportion of the analysed plasmids (57 %) carrying both BMRGs and ARGs were conjugative whereas only 18 % of plasmids without co-selection potential (i.e. plasmids with only BMRGs, only ARGs or no resistance genes at all) were conjugative (Fig. 7a). Thus, plasmids with co-selection potential tend to be large and conjugative. Also when controlling for the effect of size, plasmids carrying both types of resistance genes were more often conjugative (logistic regression, 14.4 % versus 2.6 %; *p* < 0.0001). Importantly, bacterial isolates from polluted environments carried notably higher proportion of plasmids with resistance genes and conjugation systems (Additional file 1: Table S4).

### Plasmids carrying BMRGs are more likely to carry toxin-antitoxin systems

Plasmids with only BMRGs (*p* < 0.0001) or with both BMRGs and ARGs together (*p* < 0.0001) more frequently carried toxin-antitoxin systems than did plasmids without any resistance genes (Fisher’s exact test) (Fig. 7b). This is largely explained by BMRGs and toxin-antitoxin systems both being more frequent with larger plasmids size (Fig. 5 and Additional file 1: Figure S9). However, plasmids with only ARGs did not follow a similar size distribution (Fig. 5). Accordingly, toxin-antitoxin systems were equally common on plasmids carrying only ARGs as on plasmids carrying no resistance genes at all (28 % versus 26 %; Fisher’s exact test, *p* = 0.3436).

### Metals and QACs are potential co-selectors of ARGs

**Fig. 8:**
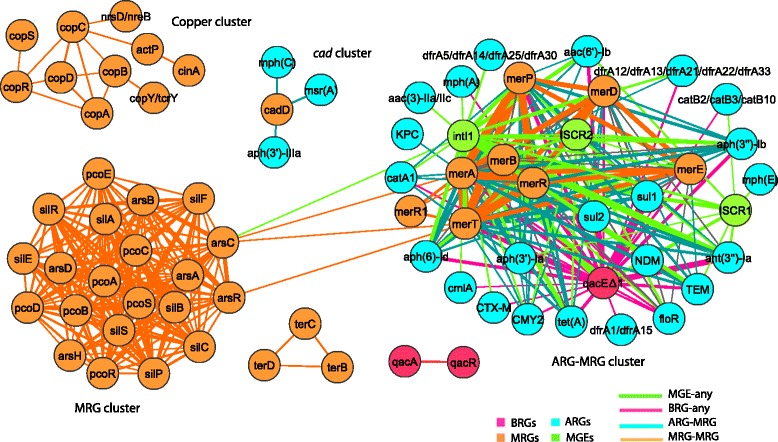
Co-occurrence network of resistance genes on plasmids. The network was built based on the observed co-occurrence pattern of BMRGs with ARGs on 4582 plasmids. The network was filtered such that only connections between BMRGs and ARGs, and between integrases/transposases and resistance genes were kept if the connected genes occurred together on at least 10 plasmids. No connections are shown between ARGs to better emphasize the co-selection potentials. The thickness of each connection (edge) between two resistance genes (nodes) is proportional to the number of times the two resistance genes co-occurred on the same plasmids

To investigate the co-occurrence of BMRGs and ARGs on the same plasmids, a network of resistance genes was generated. In total, six distinct resistance gene clusters were observed and two of them were lightly connected (Fig. 8). An isolated cluster (the ‘*cad* cluster’) contained a resistance gene (*cadD*) towards cadmium and zinc, co-occurring with resistance genes towards aminoglycosides and macrolides. Another distinctly isolated copper resistance gene cluster (the ‘copper cluster’) consisted mainly of resistance genes that are part of the *cop* operon together with the nickel and cobalt resistance gene *nrsD/nreB*, but no ARGs. Moreover, another set of metal resistance genes conferring resistance to arsenic, copper and silver (the ‘MRG cluster’), was strongly interconnected via three resistance gene operons (*ars, pco* and *sil*), but clustered separately from ARGs. The ‘MRG cluster’ was lightly connected, via two arsenic resistance genes, to a large cluster (the ‘ARG-BMRG cluster’) of resistance genes towards metals, biocides and antibiotics, and MGEs such as *intI1* and *ISCR* transposases. The arsenic resistance genes were specifically connected to mercury resistance genes and the class 1 integrase (*intI1)* of the large cluster.

In the ‘ARG-BMRG cluster’, mercury resistance genes, and *qacE∆1* that confers low-level resistance towards multiple chemical classes of antibacterial biocides, were strongly connected to a range of different ARGs conferring resistance to a range of different antibiotic classes (for more details see the heat map and the bar graph in Additional file 5: Figure S10 and Additional file 1: Figure S11, respectively). There was also a strong association of BMRGs and/or ARGs with MGEs, such as *intI1* and transposases including *ISCR1* and *ISCR2.* For the integrases, *qacE∆1*, mercury resistance genes and resistance genes towards aminoglycosides frequently occurred with *intI1* (Additional file 1: Figure S12). For the transposases, *qacE∆1* and resistance genes to sulfonamides and aminoglycosides frequently occurred together with *ISCR1*, whereas resistance genes to sulfonamides, amphenicols, tetracyclines, and mercury were highly frequent with *ISCR2* (Additional file 1: Figure S13).

**Fig. 9:**
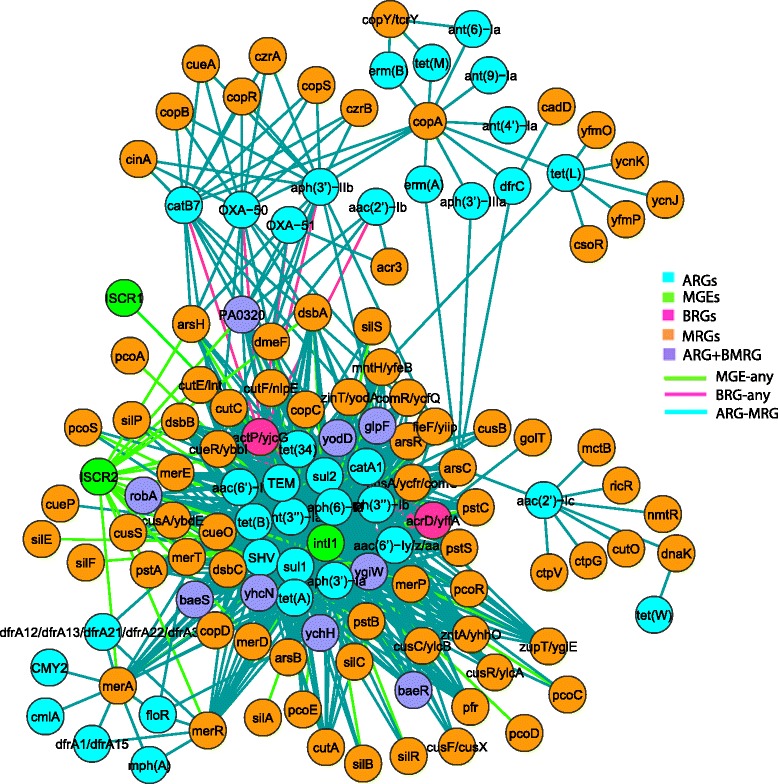
Co-occurrence network of resistance genes in genomes. The network was built based on the observed co-occurrence pattern of BMRGs with ARGs in 2522 completely sequenced bacterial genomes (2666 chromosomes and 1926 plasmids). For BMRGs, only a subset of resistance genes towards metals (i.e. mercury, arsenic, cadmium, copper, zinc, bismuth, antimony and silver) was used in order to simplify the network and make trends visible. The network was filtered such that only connections between BMRGs and ARGs, and between integrases/transposases and resistance genes were kept if the connected genes occurred together in at least ten genomes (i.e. strains) irrespective of their location (chromosomes and/or plasmids). For clarity and illustration purposes, the connections between BMRGs are not shown. No connections are shown between ARGs to better emphasize the co-selection potentials. The thickness of each connection (an edge) between two resistance genes (nodes) is proportional to the number of times the two resistance genes co-occurred in the same genomes. ‘ARG + BMRG’ refers to the genes that confer resistance/tolerance to both antibiotics and biocides/metals

Another network was built to investigate the co-occurrence of BMRGs and ARGs in the same bacterial strain irrespective of their location on chromosome or plasmid (Fig. 9; for more details see the heat map in Additional file 6: Figure S14). In this case, resistance genes were not as distinctly clustered as for the plasmids. Some additional biocide resistance genes conferring resistance to different chemical classes of biocides such as acids, alcohols and peroxides co-occurred with resistance genes to a range of antibiotics, such as aminoglycosides, beta-lactams, tetracyclines, sulfonamides and amphenicols. Interestingly, in genomes resistance genes towards arsenic, copper, mercury and silver were connected with many ARGs, in contrast to the situation on plasmids.

## Discussion

In this study, we present new insights into the co-selection potential of antibacterial biocides and metals towards antibiotics, based on common co-occurrences of resistance genes. With regards to plasmids, and hence increased risks for HGT, mercury and QACs provide a widespread co-selection potential for different antibiotic classes in bacteria of human and domestic animal origin. In contrast, resistance genes to most other biocides and metals, including silver and copper, rarely occurred together with ARGs on plasmids, suggesting a more limited risk scenario. However, BMRGs were commonly found on bacterial chromosomes from all environments. Based on this, we propose that the most common co-selection scenario between classes of chemicals is likely mediated via chromosomal BMRGs and plasmid-borne ARGs present in the same strain. This type of co-occurrence is, however, not likely to directly promote HGT. Certain bacterial taxa comprising many pathogens were particularly prone to carrying both BMRGS and ARGs, highlighting the potential clinical consequences of co-selection. We also showed that plasmids with high co-selection potential were significantly more often conjugative than were plasmids not carrying both types of resistance genes. Such ability to independently move between hosts highlights the risk for resistance transfer between strains, species and environments. Finally, the majority of the plasmids with BMRGs also carried toxin-antitoxin systems. This is expected to increase their longevity in bacterial populations even in the absence of selection pressure by antibiotics, biocides or metals.

### Co-selection potential of biocides and metals is specific towards certain antibiotics

In the network of plasmid-borne resistance genes, arsenic, copper and silver resistance genes clustered separately from ARGs. Thus, these metals have higher potential to select for resistance towards each other, but more rarely so for antibiotics. The ‘ARG-BMRG cluster’ suggests that mercury and QACs are particularly likely to co-select for antibiotic resistance to a range of different classes of antibiotics. Although mercury compounds are nowadays rarely used in clinical settings and in agriculture, resistance genes to mercury are frequently found together with ARGs on plasmids, especially on plasmids with class 1 integrons and transposons [28]. Recently, a correlation between environmental mercury exposure and increased antibiotic resistance has been observed in *E. coli* isolates from different human populations [29]. Furthermore, 25 % of some 800 antibiotic-resistance plasmids isolated from clinical *E. coli* strains carried mercury resistance determinants [30]. Genetic co-occurrences and empirical observations together provide indications that mercury can promote resistance to a wide range of antibiotics.

MGEs such as integrons and *ISCR* transposons were often found on the same plasmids as ARGs and BMRGs. Thus, selective pressures favouring maintenance of BMRGs within gene-cassettes of integrons may contribute to the maintenance of ARGs that are physically linked. Previous research has found that *qac* resistance genes and class 1 integrons are more prevalent in bacteria exposed to detergents, biocides and/or antibiotics, and *qacE*Δ*1* often occur within the class 1 integron gene-cassettes [31]. Thus, QACs may act as major selective agents for promotion of class 1 integrons [32]. Such empirical evidence and our findings together suggest that transposons and integrons play a role in the process of biocide/metal-driven co-selection of antibiotic resistance. Class 1 integrons are commonly found among pathogens, although many environmental variants of *intI1* have also been detected in environmental bacterial communities, mainly affected by anthropogenic activities [33–35]. Thus, biocide/metal-driven selection can promote increases in abundance of integrons as well as their induction, potentially facilitating acquisition of ARGs from environmental bacteria to clinical pathogens. Some studies have suggested that metal resistance genes were carried on MGEs such as integrons and transposons in bacteria from the ‘pre-antibiotic era’ [36, 37].

The observed ‘cad cluster’ is to our best knowledge the first report on a plasmid co-localization of a gene conferring resistance to cadmium/zinc (*cadD*) and resistance genes to aminoglycosides and macrolides. This genetic co-localization suggests that these heavy metals could not only to co-select for resistance towards aminoglycosides and macrolides but also promote HGT. Zinc/cadmium supplementation is commonly used for growth promotion in animals in some regions [38, 39]. Thus, unrestricted use of these metals could potentially promote antibiotic resistance towards macrolides and aminoglycosides. Additional co-occurrence patterns between BMRGs and ARGs on plasmids have been reported [40–45], and many of them were observed in our analysis, but were not highly frequent.

Co-occurrences of resistance genes within the same bacterial strain, but not necessarily on plasmids, revealed considerably denser and widespread interconnection between BMRGs and ARGs. Such co-occurrences imply that a selection pressure for maintaining one of them will aid in dissemination and increase of the relative abundance also of the other. This has the potential to increase longevity of these genes in the environment, and act against their elimination from bacterial genomes. Overall, we conclude that copper, silver, arsenic, antimony, cobalt, nickel, cadmium, iron, zinc, mercury and QACs are all potential co-selectors for strains resistant to, e.g. sulfonamides, beta-lactams, amphenicols, tetracyclines and aminoglycosides.

### Co-selection potential is higher in clinically important taxonomic groups

Presence of ARGs on plasmids was particularly frequent in clinically important bacterial genera. BMRGs were found in a wider variety of bacteria, but co-occurrence was thus, logically, mainly restricted to the clinically important ones. This suggests that selection pressure from antibacterial biocides/metals could pose a direct risk for co-selection of antibiotic resistance in clinical settings. Many plasmids with potential for co-selection were also found in uncultured bacteria (Fig. 2), suggesting that there might be many more bacteria, still uncultivable by standard microbiology techniques, which have the potential for co-selection. Overall, we conclude that although plasmids are found in wide range of bacterial genera, plasmids with ARGs and/or BMRGs are more common in certain bacterial taxa than others. One must, however, bear in mind that the completely sequenced plasmids so far unevenly represent the phylogenetic tree of bacteria, but still cover a wide range of bacterial taxa from environmental bacteria to clinically relevant species (Additional file 1: Figure S2 and S6), thus supporting the generalizations made here.

### Bacteria isolated from human and domestic animal origins have the highest co-selection potential

Humans and domestic animals carried a higher proportion of strains with co-selection potential compared to other environments. The same holds true also when only considering plasmids. Most likely, this is a logical consequence of that these are the two environments where ARGs are most common, whereas BMRGs are more widely distributed across different milieus. We therefore hypothesize that it is mainly exposure to antibiotics that has driven the overrepresentation of co-occurrences between ARGs and BMRGs in these environments, rather than exposure to biocides or metals. This fits well with that microbiota from humans and domestic animals are the only two environments regularly and intentionally exposed to high levels of antibiotics. That said, use of antibacterial products such as disinfectants, antiseptics and other metal-based products in health care and animal farming is widespread [38, 46–48] although we could not see as clear and general increases of BMRGs in isolates from such environments as we did for ARGs. Biocides and metals may, however, still contribute to why plasmids carrying genes conferring resistance to multiple chemical agents (e.g. antibiotics, biocides and metals) have recently been isolated from many hospital environments [49–51].

Isolates from some sources (polluted, plants, food and soils) showed a tendency to carry high number of biocide/metal resistance plasmids (but without ARGs). One could speculate that this is a reflection of exposure to antibacterial biocides and metals in these environments. Obviously, a selection pressure caused by the use of biocides and metals would be expected to promote resistance development to those compounds as has been proposed in several studies [52–55]. However, it should be recalled that in many environments, metals are naturally occurring at high concentrations, favouring bacteria that carry resistance/tolerance mechanisms. Also, given the often broad substrate specificity of biocide resistance genes, naturally occurring substances could very well play a major role in their maintenance.

### BMRGs are common and chromosomal BMRGs have co-selection potential

Metal tolerance/resistance is a common feature of many microorganisms dealing with metal exposure in their natural habitats. BMRGs were highly abundant, especially on chromosomes, and occurred in over 100 copies in some isolates. However, a large number of genes linked to tolerance/resistance mechanisms are common within the genomes of some bacteria. An example is *Janthinobacterium sp. HH01,* where altogether more than 2.4 % (170 chromosomal genes) of the genome has been reported to confer tolerance or resistance towards biocides/metals and antibiotics [56]. Some of those resistance or tolerance genes serve in the basic defence of the cell against superfluous metals, but some are highly specialized and are known to occur only in a few species.

The majority of bacterial chromosomes (85 %) carried genes involved in biocide/metal resistance mechanisms, some of which were ubiquitous across all environments. There was also an association between presence of BMRGs and ARGs (Fig. 1d and e), although plasmids usually carried more ARGs than BMRGs in the genomes of pathogens (Additional file 1: Figure S8). Thus, we predict that a common co-occurrence combination of resistance genes across classes would be represented by chromosomal BMRGs and plasmid-borne ARGs. This kind of co-selection scenario was frequently observed in many bacterial genera encompassing pathogens. Occurrence of ARGs and BMRGs on a plasmid would be a high-risk scenario for dissemination, because via HGT, recipient bacteria will acquire ARGs as well as BMRGs that can quickly improve the fitness of an organism in an ecosystem where there is a selection pressure by metals, biocides or antibiotics. Importantly, unless both types of genes are located on a genetic element that is horizontally transferrable, such as a plasmid, co-selection will stop following an HGT event (of the plasmid). On the other hand, with an increased use and hence selection pressure from biocides and metals, we might see further mobilization of resistance genes and operons into plasmids in the future.

Importantly, we may have most likely underestimated the presence of chromosomal resistance genes, as non-transferrable chromosomal genes are generally more variable than transferable genes [15]. Thus, given the databases at hand, the challenge to identify them as functional resistance genes is harder.

### Bacteria and plasmids without resistance genes

Over 70 % of plasmids and 14 % of all genomes lacked known resistance genes, and aquatic environments, symbiotic strains, as well as bacteria isolated from extreme environments carried low average numbers of BMRGs per genome. Furthermore, plasmids from insect symbionts carried almost no resistance genes at all. In some of those cases, the specialized environment may pose more acute restrictions on the bacteria than do toxicants, which in turn can lead to genome streamlining, i.e. reducing the number of less important genes to keep genome size down [57]. Such ecological strategies, reducing the number of, e.g. detoxification genes, have been revealed in nutrient-poor environments, such as for marine plankton [58] and symbiotic or parasitic bacteria [59, 60], reducing even oxidative stress tolerance systems down to a bare minimum [61]. Alternatively, there could be other genes for these functions that have not yet been characterized.

### Plasmids with co-selection potential tend to be conjugative

Our estimate of 20 % conjugative plasmids, 23 % mobilizable without own conjugation systems, and over half of all plasmids non-transmissible is similar to earlier estimates by Smillie et al. [21], though an expanded set of plasmids were studied here. Interestingly, we found that plasmids with co-selection potential tend to be large and conjugative.

Many of the small resistance plasmids were non-transmissible and only carried ARGs. The lack of BMRGs can be explained by the large size of the plasmid-borne metal resistance determinants that usually occur as operons and thus cannot fit in small plasmids. Over half of the plasmids larger than 250 kb were also non-transmissible; however there is evidence of their ongoing domestication into secondary chromosomes [62, 63]. Plasmid size distribution of conjugative and mobilizable plasmids has been predicted to be conserved [64]. Moreover, it is possible for conjugative plasmids to mobilize small non-transmissible plasmids with ARGs [65]. Thus, the risk for dissemination of ARGs is not limited to conjugative plasmids. In other words, selection pressure from biocides or metals in different environments has the potential to increase risks for conjugative resistance plasmid transfer from harmless bacteria to pathogens. Thereby, the risk for dissemination of ARGs due to biocides/metal-driven co-selection is not limited to certain ecological boundaries, and conjugative plasmids have roles in dissemination of ARGs across environments, given sufficient ecological connectivity between them.

### Plasmids with co-selection potential tend to carry toxin-antitoxin systems

Plasmids carrying BMRGs (alone or in combination with ARGs) more often carried toxin-antitoxin systems than did plasmids without BMRGs. Many types of genes, including BMRGs and those involved in toxin-antitoxin systems, are likely to be more common on larger plasmids, simply because larger plasmids carry more genes. Thus, the observed pattern may be rather coincidental rather than a result of intricate co-evolution. Nevertheless, the anticipated effect provided by toxin-antitoxin systems most likely affects persistence regardless of plasmid size. Hence, their co-existence is likely to contribute to the overall risk of promoting and maintaining resistance plasmids with co-selection potential.

## Conclusions

Though there are numerous case studies and reports of co-selection for antibiotic resistance by biocides and metals [5, 43, 54], our study is the first large-scale attempt to gather a broader understanding of their co-selection potential in bacteria. By systematic genomic analyses, our study provides a considerably more comprehensive picture than has previously been described. Screening of all bacterial genomes and plasmids revealed surprisingly few previously unknown connections between resistance genes to antibiotics, biocides and metals. Thus, the genetic landscape of known plasmids described in this study shows that there are limited opportunities for biocides and metals to promote HGT of antibiotic resistance, whereas there are ample possibilities for these chemicals to select for antibiotic-resistant bacteria through chromosomal BMRGs. We believe that our results enhance our understanding the underlying genetics of co-selection and put us in a better position to evaluate the possible risk scenarios of resistance dissemination in the future, thereby facilitating better management of certain aspects of antibiotic resistance development. In the future, we would encourage more empirical experiments designed to investigate to what extent the genetic connections are reflected in actual co-selective ability. Furthermore, large-scale studies on mutation-based resistance [66, 67] as well as cross-resistance [68] would provide a more complete picture, as these mechanisms are expected to act in parallel to co-selection via co-occurrence of resistance genes. It should be noted that the fully sequenced isolates and plasmids still only represent a minute, and partly biased, fraction of the bacteria present in different environments. Further investigation using metagenomics could therefore further aid in the identification of environments where risks for co-selection is high.

## Additional files

Additional file 1:
**Additional data file 1 contains additional tables and figures.** **Table S1**. Metadata of 2522 completely sequenced bacterial genomes comprising of 2666 chromosomes and 1926 plasmids analysed in this study (provided as Additional file 2). **Table S2**. Pfam profiles for well-described toxin-antitoxin systems. **Table S3**. Summary of the completed genomes dataset. **Table S4**. Co-selection potential of bacterial isolates from different environments and overview of plasmid mobility. **Table S5**. Summary of the plasmid mobility potential for 1926 plasmids from 2522 completed genomes. **Figure S1**. Taxonomic distribution of the 2522 completely sequenced bacterial strains analysed in this study. **Figure S2**. Taxonomic distribution of the bacteria that harboured the 4582 plasmids. **Figure S3**. Distribution of the 2522 completely sequenced bacterial genomes across environmental categories. **Figure S4**. Distribution of 1187 resistance plasmids (out of 4582 total plasmids) based on the number of resistance genes present in each plasmid. **Figure S5**. The 25 most abundant resistance genes found on plasmids and chromosomes. **Figure S6**. Top bacterial genera that hosted 4582 completely sequenced plasmids. **Figure S7**. Relative frequencies of plasmid-borne resistance genes in the top 20 bacterial genera harbouring the 4582 plasmids. **Figure S8**. Relative frequencies of resistance genes in clinically important genera. **Figure S9**. Distributions of plasmids based on toxin-antitoxin systems and resistance genes. **Figure S10**. Co-occurrences of ARGs and BMRGs on 4582 plasmids. (provided as Additional file 5) **Figure S11**. The 20 most common co-occurrence combinations of antibiotic resistance genes and biocide/metal resistance genes on 4582 plasmids. **Figure S12**. The 20 most common co-occurrences of resistance genes with integron-associated class 1 integrases on 4582 plasmids. **Figure S13**. The 20 most common co-occurrences of resistance genes with *ISCR* transposase elements on 4582 plasmids. **Figure S14**. Co-occurrences of ARGs and BMRGs in 2522 bacterial genomes. (provided as Additional file 6). (DOCX 770 kb) Additional file 2: Table S1. Metadata of 2522 completely sequenced bacterial genomes comprising of 2666 chromosomes and 1926 plasmids analysed in this study. (XLSX 331 kb) Additional file 3:
**Environmental classification of bacterial isolates.** (DOCX 24 kb) Additional file 4:
**R code for statistical analysis.** (TXT 4 kb) Additional file 5: Figure S10. Co-occurrences of ARGs and BMRGs on 4582 plasmids. (PDF 42 kb) Additional file 6: Figure S14. Co-occurrences of ARGs and BMRGs in 2522 bacterial genomes. (PDF 357 kb)

## Abbreviations

ARGs: Antibiotic Resistance Genes
BMRGs: Biocide and Metal Resistance Genes
HGT: Horizontal Gene Transfer
HMM: hidden Markov model
QACs: Quaternary Ammonium Compounds
MGEs: Mobile Genetic Elements
