# Supplementary material for: Co-occurrence of resistance genes to antibiotics, biocides and metals reveals novel insights into their co-selection potential
Source: BMC Genomics. 2015 Nov 17;16:964. doi: 10.1186/s12864-015-2153-5 (PMC4650350; doi:10.1186/s12864-015-2153-5)
Supplement: Additional file 1: — Additional data file 1 contains additional tables and figures. Table S1. Metadata of 2522 completely sequenced bacterial genomes comprising of 2666 chromosomes and 1926 plasmids analysed in this study (provided as Additional file 2). Table S2. Pfam profiles for well-described toxin-antitoxin systems. Table S3. Summary of the completed genomes dataset. Table S4. Co-selection potential of bacterial isolates from different environments and overview of plasmid mobility. Table S5. Summary of the plasmid mobility potential for 1926 plasmids from 2522 completed genomes. Figure S1. Taxonomic distribution of the 2522 completely sequenced bacterial strains analysed in this study. Figure S2. Taxonomic distribution of the bacteria that harboured the 4582 plasmids. Figure S3. Distribution of the 2522 completely sequenced bacterial genomes across environmental categories. Figure S4. Distribution of 1187 resistance plasmids (out of 4582 total plasmids) based on the number of resistance genes present in each plasmid. Figure S5. The 25 most abundant resistance genes found on plasmids and chromosomes. Figure S6. Top bacterial genera that hosted 4582 completely sequenced plasmids. Figure S7. Relative frequencies of plasmid-borne resistance genes in the top 20 bacterial genera harbouring the 4582 plasmids. Figure S8. Relative frequencies of resistance genes in clinically important genera. Figure S9. Distributions of plasmids based on toxin-antitoxin systems and resistance genes. Figure S10. Co-occurrences of ARGs and BMRGs on 4582 plasmids. (provided as Additional file 5) Figure S11. The 20 most common co-occurrence combinations of antibiotic resistance genes and biocide/metal resistance genes on 4582 plasmids. Figure S12. The 20 most common co-occurrences of resistance genes with integron-associated class 1 integrases on 4582 plasmids. Figure S13. The 20 most common co-occurrences of resistance genes with ISCR transposase elements on 4582 plasmids. Figure S14. Co-occurrences of ARGs a [file 12864_2015_2153_MOESM1_ESM.docx]

**Additional file 1: Additional Tables and Figures**

**Addition file 1: Table S1.** Metadata of 2522 completely sequenced bacterial genomes comprising of 2666 chromosomes and 1926 plasmids analysed in this study. (provided as Additional file 2) **Table S2.** Summary of the completed genomes dataset. **Table S3**. Co-selection potential of bacterial isolates from different environments and overview of plasmid mobility. **Table S4.** Summary of the plasmid mobility potential for 1926 plasmids from 2522 completed genomes. **Figure S1**. Taxonomic distribution of the 2522 completely sequenced bacterial strains analysed in this study. **Figure S2.** Taxonomic distribution of the bacteria that harboured the 4582 plasmids. **Figure S3.** Distribution of the 2522 completely sequenced bacterial genomes across environmental categories**. Figure S4.** Distribution of 1187 resistance plasmids (out of 4582 total plasmids) based on the number of resistance genes present in each plasmid. **Figure S5.** The 25 most abundant resistance genes found on plasmids and chromosomes. **Figure S6.** Top bacterial genera that hosted 4582 completely sequenced plasmids. **Figure S7.** Relative frequencies of plasmid-borne resistance genes in the top 20 bacterial genera harbouring the 4582 plasmids. **Figure S8.** Relative frequencies of resistance genes in clinically important genera. **Figure S9**. Distributions of plasmids based on toxin-antitoxin systems and resistance genes. **Figure S10**. Co-occurrences of ARGs and BMRGs on 4582 plasmids. (provided as Additional file 5) **Figure S11.** The 20 most common co-occurrence combinations of antibiotic resistance genes and biocide/metal resistance genes on 4582 plasmids. **Figure S12.** The 20 most common co-occurrences of resistance genes with integron-associated class 1 integrases on the same plasmids. **Figure S13**. The 20 most common co-occurrences of resistance genes with *ISCR* transposase elements. **Figure S14.** Co-occurrences of ARGs and BMRGs in 2522 bacterial genomes. (provided as Additional file 6)

**Table S1.** Metadata of 2522 completely sequenced bacterial genomes comprising of 2666 chromosomes and 1926 plasmids analysed in this study.

[Table S1 (format: .xlsx) has been provided as a separate file (Additional file 2) as it is too large to integrate here.]

**Table S2.** Summary of the completed genomes dataset

| Number of completely sequenced (full) genomes | 2522 |
| --- | --- |
| Number of plasmids present in the full genomes | 1926 |
| Number of chromosomes present in the full genomes | 2666 |
| Number of genomes with plasmids | 843 |
| Number of genomes with resistance plasmids | 274 |

**Table S3**. Co-selection potential of bacterial isolates from different environments and overview of plasmid mobility

| Environments | Genomes with both ARGs and BMRGs (%) | Plasmids with both BMRGs and ARGs (%) | Resistance plasmids (%) | Conjugative plasmids (%) | Plasmids with resistance genes and conjugation systems (%) |
| --- | --- | --- | --- | --- | --- |
| Aquatic | 5.4 | 0 | 17.6 | 31.2 | 7.1 |
| Extreme | 1.6 | 0 | 10.7 | 17.0 | 3.6 |
| Polluted | 9.0 | 0 | 35.3 | 38.2 | 21.3 |
| Soils | 8.4 | 0.3 | 18.6 | 22.9 | 6.3 |
| Plants | 11.2 | 0.7 | 29.7 | 31.2 | 8.0 |
| Food | 10.8 | 0.6 | 29.7 | 16.9 | 8.7 |
| Symbionts | 0.7 | 0 | 1.5 | 3.7 | 0 |
| Humans | 30.9 | 5.4 | 18.7 | 17.7 | 5.8 |
| Domestic animals | 21.5 | 7.0 | 20.8 | 30.7 | 8.0 |
| Wild animals | 7.6 | 0 | 3.3 | 15 | 1.7 |
| Lab/engineered | 29.1 | 1.9 | 15.4 | 17.3 | 3.8 |
| Unknown | 18.9 | 0 | 24.1 | 13.8 | 6.9 |
| **Overall %** | **17.0** | **2.0** | **19.5** | **21.5** | **6.9** |

**Table S4.** Summary of the plasmid mobility potential for 1926 plasmids from 2522 completed genomes

| Isolation source | Non-transmissible | Mobilizable | Conjugative |
| --- | --- | --- | --- |
| Aquatic | 86 | 31 | 53 |
| Extreme | 75 | 19 | 19 |
| Polluted | 61 | 23 | 52 |
| Soils | 153 | 82 | 69 |
| Plants | 60 | 25 | 43 |
| Food | 69 | 79 | 29 |
| Symbionts | 122 | 9 | 5 |
| Humans | 312 | 113 | 92 |
| Domestic animals | 58 | 23 | 31 |
| Wild animals | 25 | 19 | 9 |
| Lab/engineered | 23 | 19 | 9 |
| Unknown | 18 | 7 | 4 |
| TOTAL (1926 plasmids) | **1062** | **449** | 415 |
| Overall % | 55.1 | 23.3 | 21.6 |


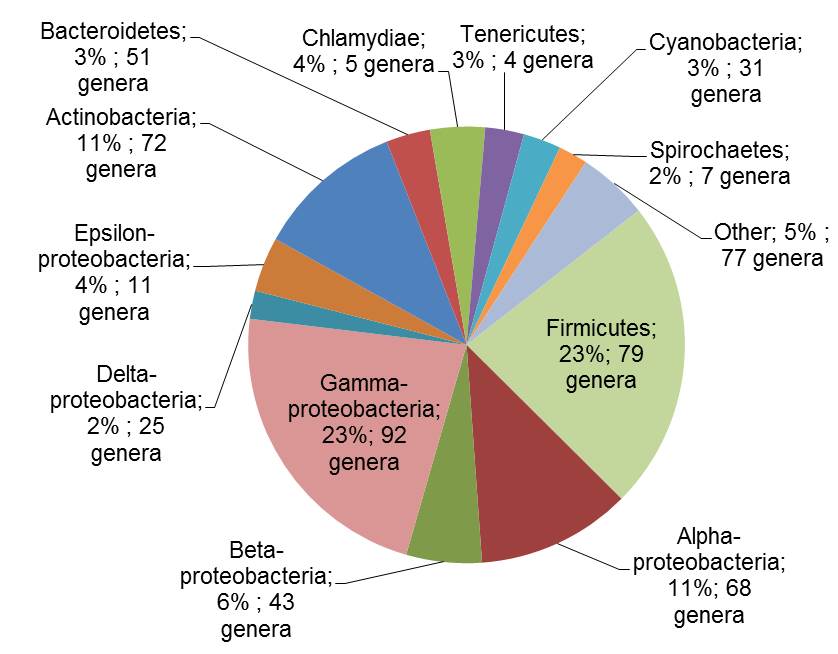


**Figure S1**. **Taxonomic distribution of the 2522 completely sequenced bacterial strains analysed in this study**. Only the phylum Proteobacteria was further classified to class level. Each individual section of the pie chart indicates the percentage of the total genomes carried within the phylum/class and the number of corresponding genera.


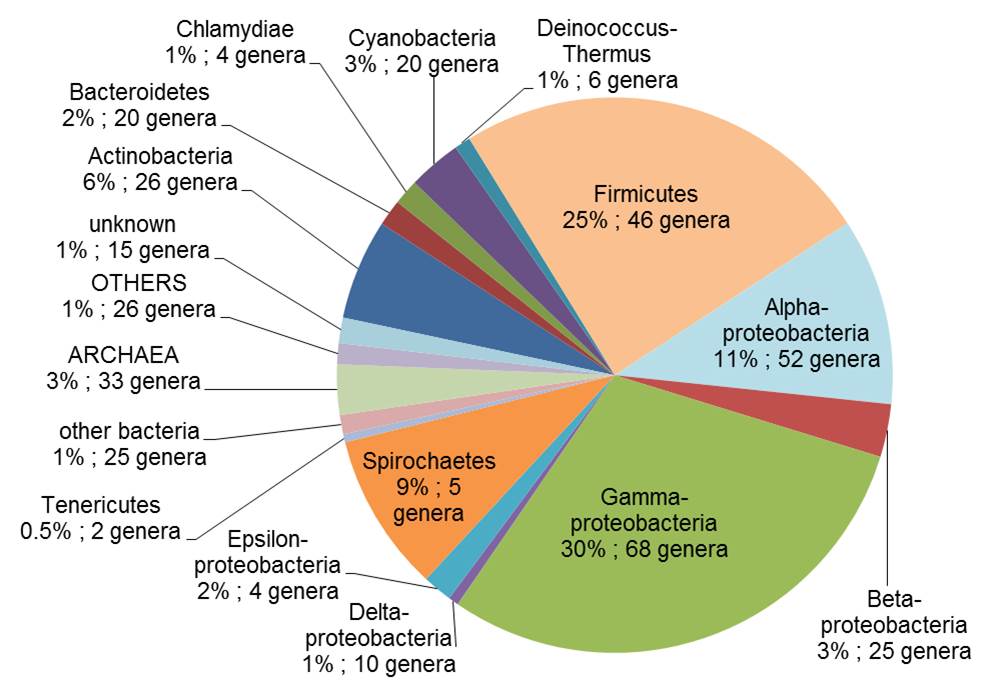


**Figure S2.** **Taxonomic distribution of the bacteria that harboured the 4582 plasmids.** Only the phylum Proteobacteria was further classified to class level. Each individual section of the pie chart indicates the percentage of the total plasmids stemming from the phylum/class and the number of corresponding genera that carried those plasmids.

**
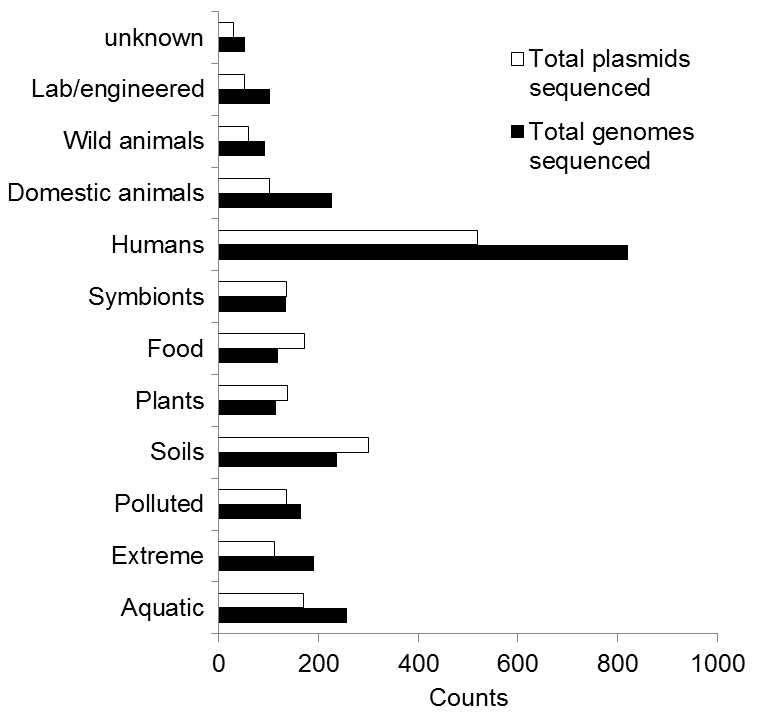
**

**Figure S3. Distribution of the 2522 completely sequenced bacterial genomes across environmental categories.** The black bars represent the number of genomes analysed from each environment and the white bars represent the number of plasmids within the genomes from each environmental category.


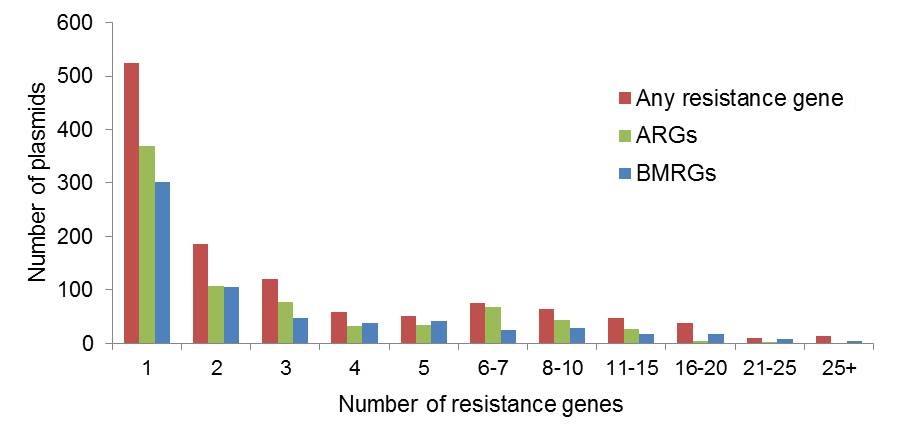


**Figure S4**. **Distribution of 1187 resistance plasmids (out of 4582 total plasmids) based on the number of resistance genes present in each plasmid.** Each bar represents the number of plasmids carrying certain number of resistance genes (1 to over 25 resistance genes) in each individual plasmid.





**Figure S5.** **The 25 most abundant resistance genes found on plasmids and chromosomes.** **(a)** ARGs on 4582 plasmids. **(b)** BMRGs on 4582 plasmids. **(c)** ARGs on 2666 chromosomes from 2522 genomes and **(d)** BMRGs on 2666 chromosomes from 2522 genomes.


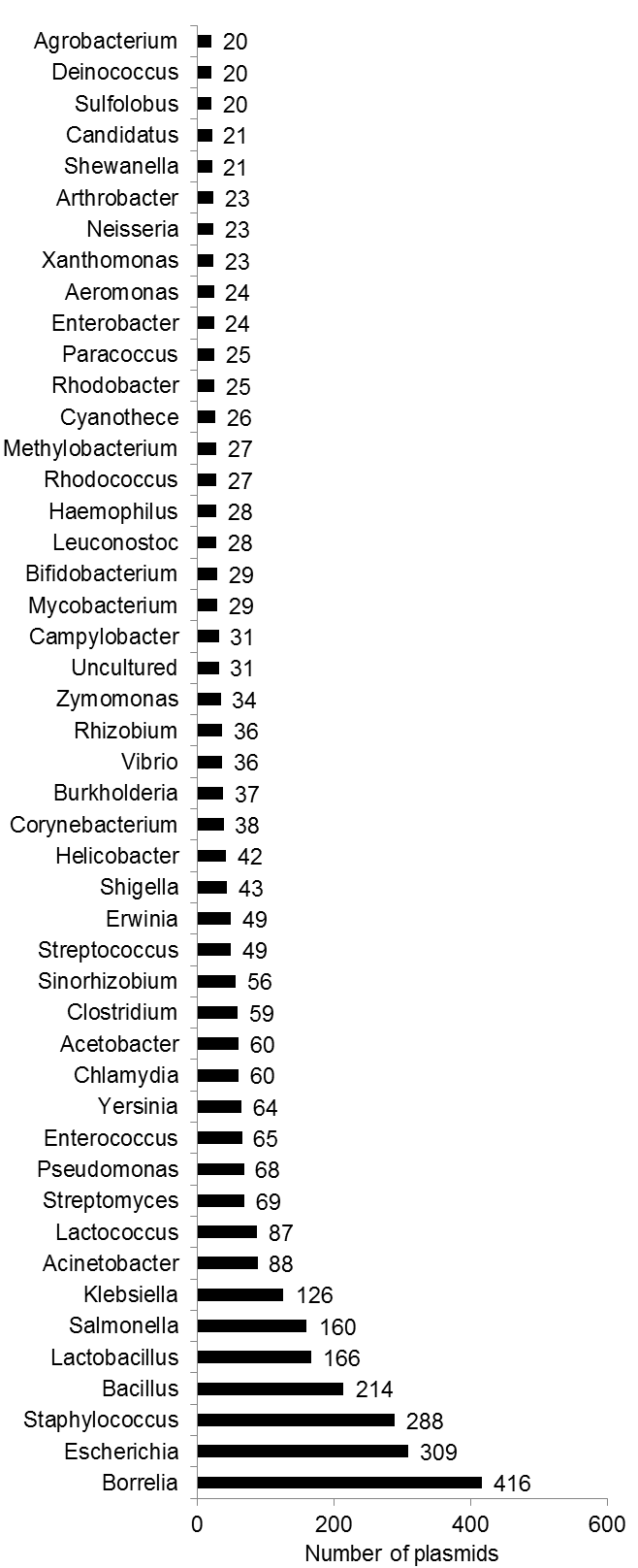


**Figure S6. Top bacterial genera that hosted 4582 completely sequenced plasmids.** The figure shows only the bacterial genera that hosted at least 20 plasmids.

**(b)**

**(a)**


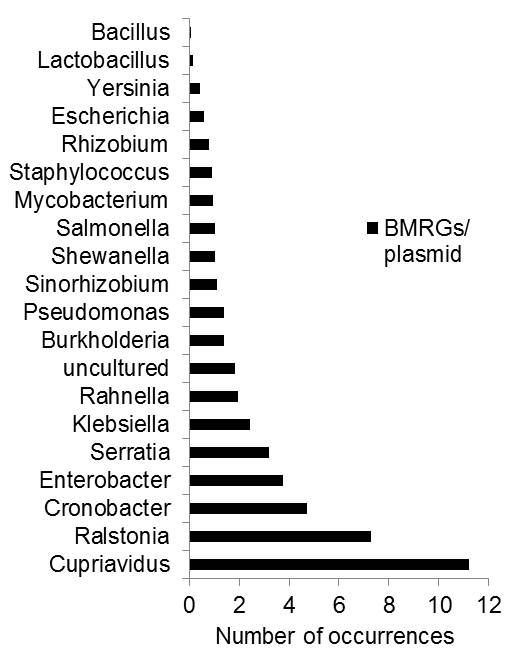

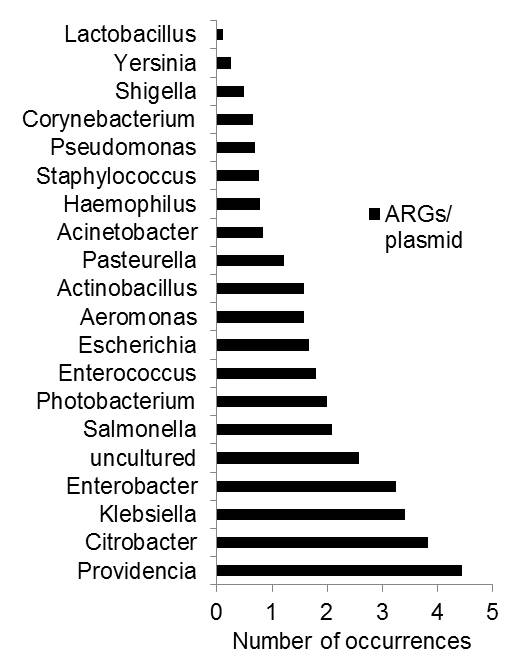


**Figure S7. Relative frequencies of plasmid-borne resistance genes in the top 20 bacterial genera harbouring the 4582 plasmids.** **(a)** Average biocide/metal resistance genes (BMRGs) present per plasmid, **(b)** Average antibiotic resistance genes (ARGs) present per plasmid.


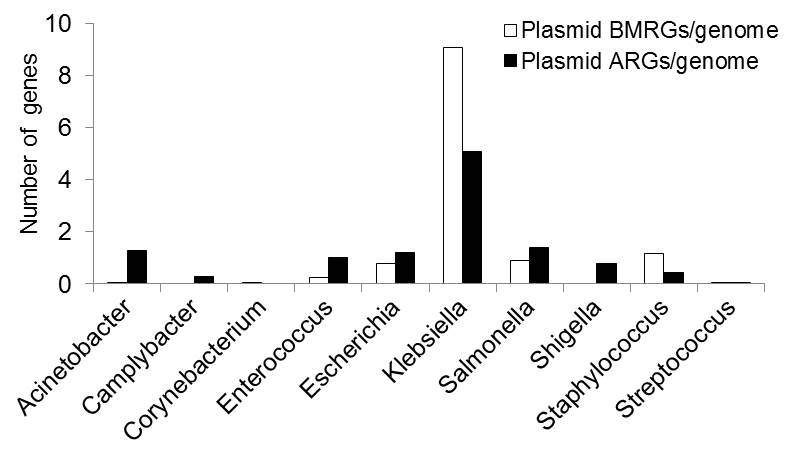


**Figure S8. Relative frequencies of resistance genes in clinically important genera.** Average numbers of plasmid-borne antibiotic resistance genes (ARGs) and biocide/metal resistance genes (BMRGs) per genome in 10 clinically important bacterial genera where the highest numbers of resistance plasmids were found.


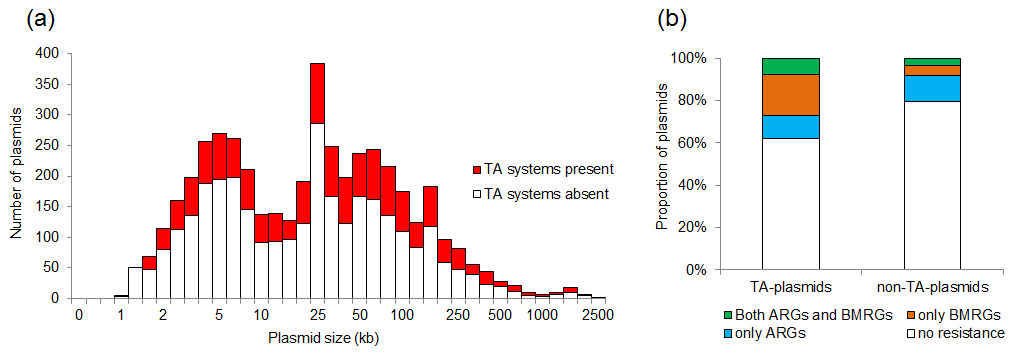


**Figure S9**. **Distributions of plasmids based on toxin-antitoxin systems and resistance genes.** (a) Distribution of plasmids according to their size with and without toxin-antitoxin systems. (b) Overview of resistance genes patterns on plasmids with and without toxin-antitoxin systems. TA-plasmids refer to plasmids carrying toxin-antitoxin (TA) systems; non-TA-plasmids refer to plasmids without TA systems;

[Figure S10 (format: .pdf) has been provided as a separate file (Additional file 5) as it is too large to integrate here]

**Figure S10.** **Co-occurrences of ARGs and BMRGs on 4582 plasmids.** Co-occurrences of biocide/metal resistance genes (BMRGs) (y-axis) with antibiotic resistance genes (ARGs) (x-axis) on the same plasmids. The colour intensity in each panel indicates frequency of co-occurrence.


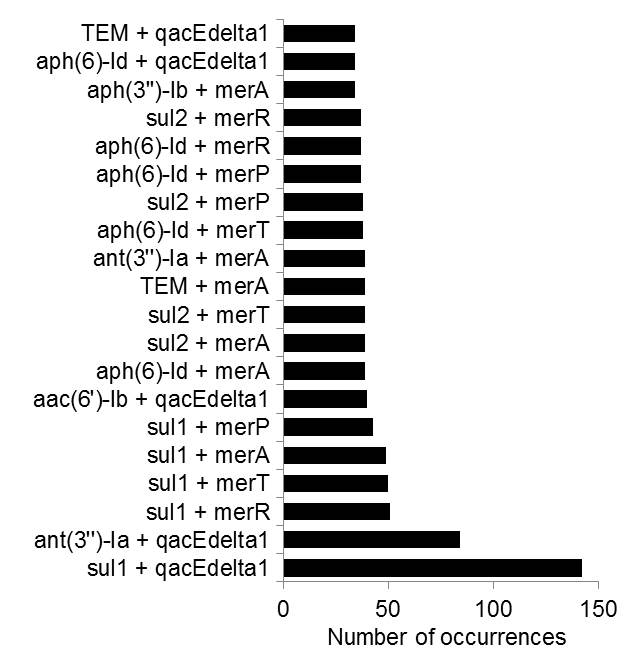


**Figure S11.** The 20 most common co-occurrence combinations of antibiotic resistance genes and biocide/metal resistance genes on 4582 plasmids.


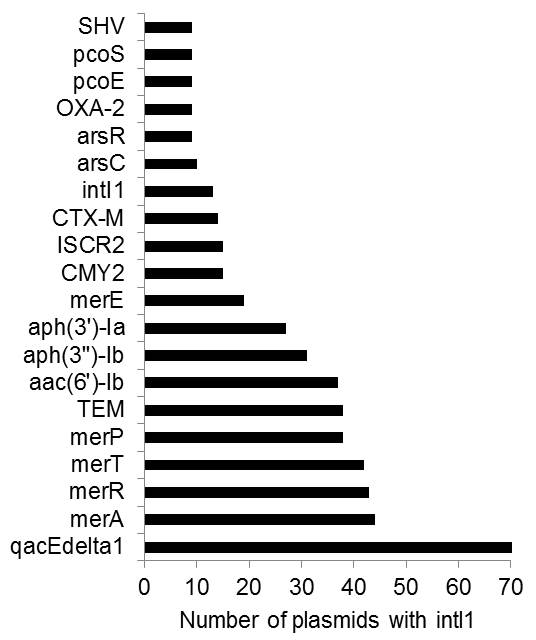


**Figure S12.** **The 20 most common co-occurrences of resistance genes with integron-associated class 1 integrases on the same plasmids.** Number of occurrences of different antibiotic and biocide/metal resistance genes that co-occur with integron-associated class 1 integrases (*intI1*) on the same plasmids (found among the set of 4582 plasmids).

**(a)**

**(b)**

(
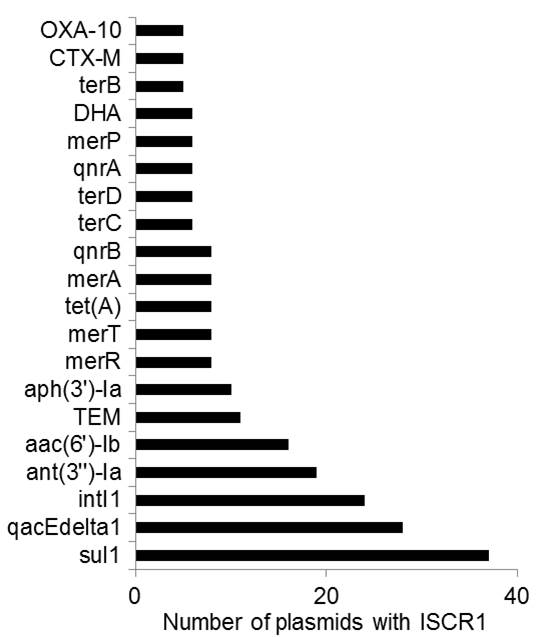

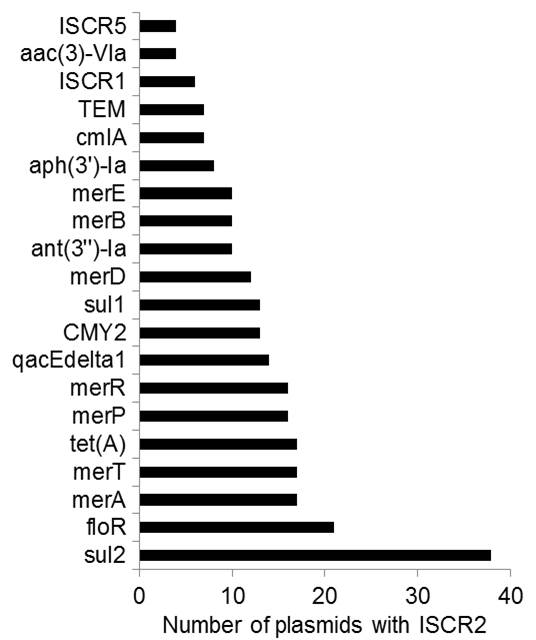


**Figure S13**. **The 20 most common co-occurrences of resistance genes with *ISCR* transposase elements.** Number of occurrences of different antibiotic and biocide/metal resistance genes that co-occur with **(a)** *ISCR1* transposase element and **(b)** *ISCR2* transposase element, on the same plasmids, found among 4582 plasmids.

[Figure S14 (format: .pdf) has been provided as a separate file (Additional file 6) as it is too large to integrate here.]

**Figure S14.** **Co-occurrences of ARGs and BMRGs in 2522 bacterial genomes**. Co-occurrences of biocide/metal resistance genes (BMRGs) (y-axis) with antibiotic resistance genes (ARGs) (x-axis) in the same bacterial strain irrespective of their location on chromosome or plasmid. The colour intensity in each panel indicates how frequently they co-occur.
