# Supplementary material for: Co-occurrence of resistance genes to antibiotics, biocides and metals reveals novel insights into their co-selection potential
Source: BMC Genomics. 2015 Nov 17;16:964. doi: 10.1186/s12864-015-2153-5 (PMC4650350; doi:10.1186/s12864-015-2153-5)
Supplement: Additional file 3: — Environmental classification of bacterial isolates. (DOCX 24 kb) [file 12864_2015_2153_MOESM3_ESM.docx]

**Additional file 3: Environmental classification of bacterial isolates**

The bacterial isolates were categorized into 12 different environments based on their isolation source. Criteria and some examples have been shown below:

1. **Aquatic**: Bacterial strains isolated from aquatic environments. For examples, isolates from groundwater, lagoons, sea water, surface water, sand coastline etc.; seaweed, algae-associated isolates from aquatic coasts, Sea beach, bay, mud, water, marine sediment, lake, river etc. have been part of this category.
2. **Extreme**: Bacteria isolated from natural extreme environments with no indication of anthropogenic impacts. For examples, hot springs, coal/metal mines, geothermal activities, Antarctic ice core etc.
3. **Polluted**: Bacterial isolates from polluted environments. Any indication of pollution by anthropogenic activities in the sample site has been considered as polluted. Some examples are contaminated soil, WWTP, alkaline compost, sewage, sludge, poultry deep litter, metal factory, chemically polluted, oil production, radioactive waste, kitchen sink, bioleaching reactors etc.
4. **Soils**: Bacterial isolates from soils or soil-associated environments (note: no records of excessive pollution in addition to amendment of fertilizers, compost, manure etc). Some examples are isolates from rhizosphere, roots, agricultural field, compost, root nodule, surface of vegetables that grown under soil (soil bacteria dominate in these sources), forest soils, cave soils, rocks etc.
5. **Plants**: Bacterial isolates from plants and tree-associated environments. Some examples are fresh grains, fresh vegetables, vegetables grown above soil, trees, plants, stems, leaves, undersoil vegetables but internal tissues etc.
6. **Food:** Bacteria isolated from food-associated environments or straight from the food. Some examples are dairy products such as yoghurt, cheese, other fermented foods etc., bacteria isolated during fermentation process (e.g. beer, wine, vinegar), fruit juice, baby milk formula, retail chicken, kimchi, sausage, ground whole grains, vitamin C production facility, food factory, isolates from food-borne outbreaks etc.
7. **Symbionts**: Bacterial isolates that live in a symbiotic relationship or live on/in insect body. Some examples are ticks, aphid, fly, mosquito, cockroach etc. Some fungal-symbiont, lichens-symbiont, amoeba-symbiont and nephridial symbionts have also been included in this category.
8. **Humans**: Bacterial isolates (including human pathogens) directly from humans or clinics. For examples, human skin, blood, gut, faeces, human milk, urine etc.
9. **Domestic animals**: Isolates from animals that have been known to be domesticated widely all over the world and have ben considered as companion animals. These are the animals that we treat with antibiotics. Some examples include cow, pig, horse, poultry, cat, dog, pet rabbit, animal milk (direct from animals) etc. Exception not included here are some wild animals that we may have treated with antibiotics such as circus lion. Additionally, isolates from aquaculture such as fish farming (only if there is an indication of farming) where antibiotics are used intentionally has been included in this category.
10. **Wild animals**: Isolates from animals, which normally are found only in nature (is not provided shelter by a human), is responsible for getting its own food and water (is not provided food or water by humans), and is not usually cared for by humans. Some examples are wild mice, mouse, rat, bird, wild monkey, kangaroo, lizard, snake and marine animals such as fish, dolphin, whale, snail shell, squid, coral etc. Some samples likely come from animals captured and held in captivity for extended times, but such information is often difficult to acquire from the metadata.
11. **Lab/engineered**: Isolates that been derived from another stain by mutagenesis or other artificial way such as genetic modification. Examples are industrial strains to produce large scale products etc.
12. **Unknown**: Isolation source is unknown in the main literature or other databases. A few isolates from very distinct environments have been put in this category as unclassified. For examples, cellulose (pulp) digester, air-borne isolate etc.
