# Supplementary material for: Co-occurrence of resistance genes to antibiotics, biocides and metals reveals novel insights into their co-selection potential
Source: BMC Genomics. 2015 Nov 17;16:964. doi: 10.1186/s12864-015-2153-5 (PMC4650350; doi:10.1186/s12864-015-2153-5)
Supplement: Additional file 5: Figure S10. — Co-occurrences of ARGs and BMRGs on 4582 plasmids. (PDF 42 kb) [file 12864_2015_2153_MOESM5_ESM.pdf]

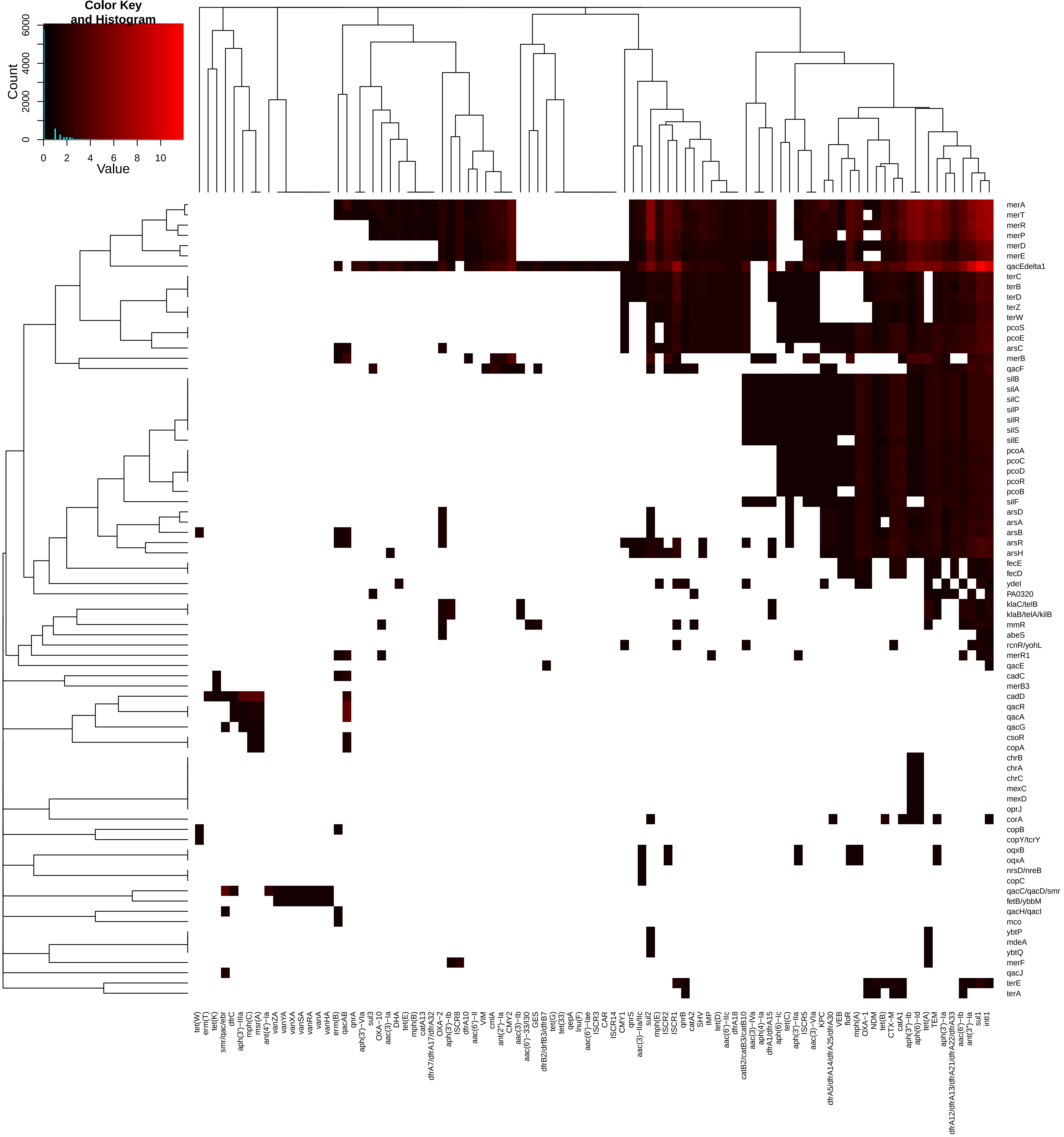

Color Key
and Histogram
Count
0
2000
4000
6000
Value
0
2
4
6
8
10
merA
merT
merR
merP
merD
merE
qacEdelta1
terC
terB
terD
terZ
terW
pcoS
pcoE
arsC
merB
qacF
silB
silA
silC
silP
silR
silS
silE
pcoA
pcoC
pcoD
pcoR
pcoB
silF
arsD
arsA
arsB
arsR
arsH
fecE
fecD
ydeI
PA0320
klaC/telB
klaB/telA/kilB
mmR
abeS
rcnR/yohL
merR1
qacE
cadC
merB3
cadD
qacR
qacA
qacG
csoR
copA
chrB
chrA
chrC
mexC
mexD
oprJ
corA
copB
copY/tcrY
oqxB
oqxA
nrsD/nreB
copC
qacC/qacD/smr
fetB/ybbM
qacH/qacI
mco
ybtP
mdeA
ybtQ
merF
qacJ
terE
terA
tet(W)
erm(T)
tet(K)
smr/qac/ebr
dfrC
aph(3')−IIIa
mph(C)
msr(A)
ant(4')−Ia
vanZA
vanYA
vanXA
vanSA
vanRA
vanA
vanHA
erm(B)
qacAB
qnrA
aph(3')−VIa
sul3
OXA−10
aac(3)−Ia
DHA
tet(E)
mph(B)
catA13
dfrA7/dfrA17/dfrA32
OXA−2
aph(3')−Ib
ISCR8
dfrA10
aac(6')−II
VIM
cmlA
ant(2'')−Ia
CMY2
aac(3)−Ib
aac(6')−33/I30
GES
dfrB2/dfrB3/dfrB7
tet(G)
tet(33)
qepA
lnu(F)
aac(6')−Iae
ISCR3
CAR
ISCR14
CMY1
qnrS
aac(3)−IIa/IIc
sul2
mph(E)
ISCR2
ISCR1
qnrB
catA2
SHV
IMP
tet(D)
aac(6')−IIc
dfrA18
catB2/catB3/catB10
aac(3)−IVa
aph(4)−Ia
dfrA1/dfrA15
aph(6)−Ic
tet(C)
aph(3')−IIa
ISCR5
aac(3)−VIa
KPC
dfrA5/dfrA14/dfrA25/dfrA30
VEB
floR
mph(A)
OXA−1
NDM
tet(B)
CTX−M
catA1
aph(3'')−Ib
aph(6)−Id
tet(A)
TEM
aph(3')−Ia
dfrA12/dfrA13/dfrA21/dfrA22/dfrA33
aac(6')−Ib
ant(3'')−Ia
sul1
intI1
