# Supplementary figures and images for: Co-occurrence of resistance genes to antibiotics, biocides and metals reveals novel insights into their co-selection potential
Source: BMC Genomics. 2015 Nov 17;16:964. doi: 10.1186/s12864-015-2153-5 (PMC4650350; doi:10.1186/s12864-015-2153-5)

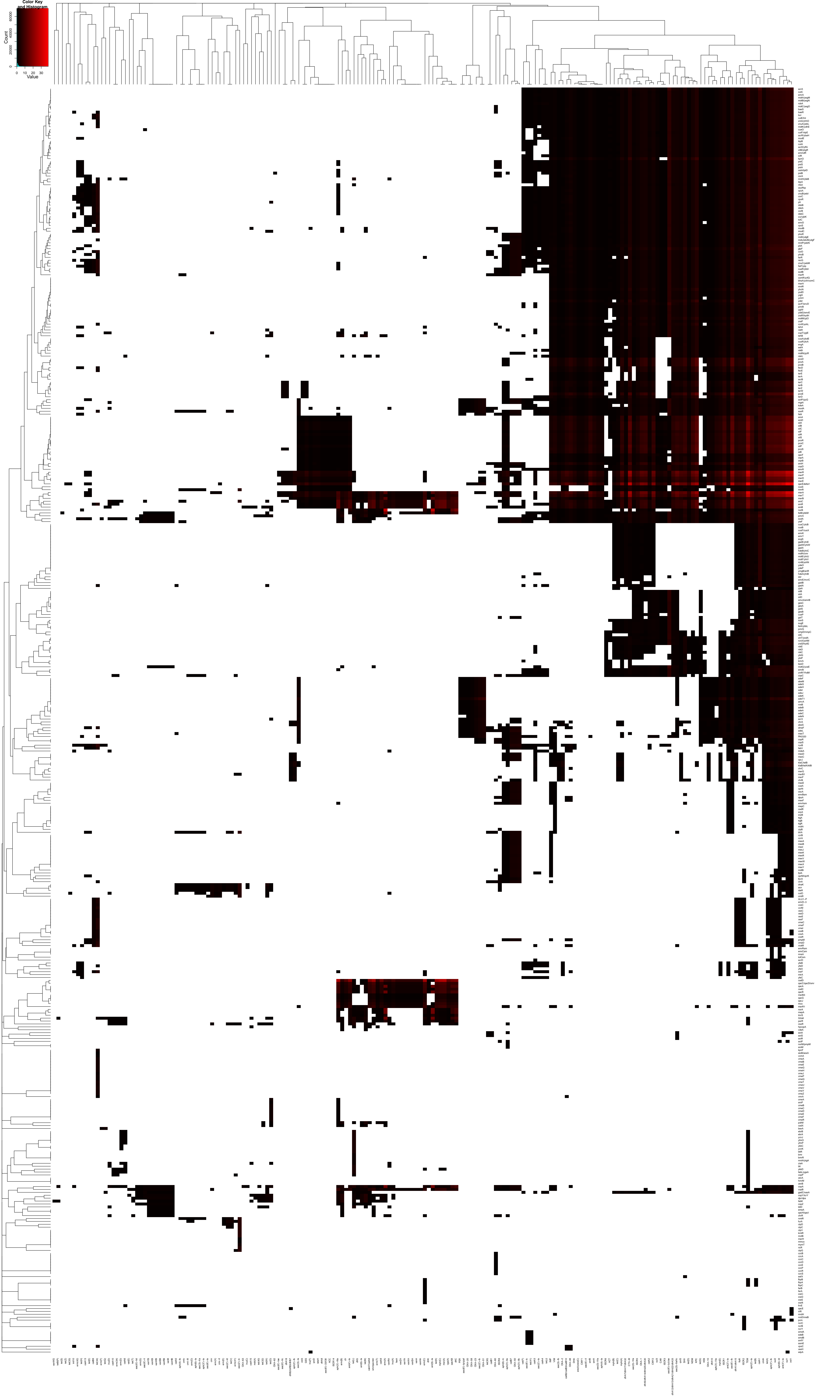

Supplement: Additional file 6: Figure S14. — Co-occurrences of ARGs and BMRGs in 2522 bacterial genomes. (PDF 357 kb) [file 12864_2015_2153_MOESM6_ESM.pdf]
